# Treatment for infections with carbapenem-resistant *Enterobacteriaceae*: what options do we still have?

**DOI:** 10.1186/cc13949

**Published:** 2014-06-27

**Authors:** Michele Yamamoto, Aurora E Pop-Vicas

**Affiliations:** 1 Department of Medicine, Memorial Hospital of Rhode Island, 111 Brewster Street, Pawtucket RI 02860, USA; 2 The Alpert Medical School of Brown University, 222 Richmond St, Providence RI 02903, USA

## Abstract

The global spread of carbapenem-resistant *Enterobacteriaceae* (CRE) is increasingly becoming a major challenge in clinical and public health settings. To date, the treatment for serious CRE infections remains difficult. The intelligent use of antimicrobials and effective infection control strategies is crucial to prevent further CRE spread. Early consultation with experts in the treatment of infections with multidrug-resistant organisms is valuable in patient management. This brief review will focus on the current, yet limited, treatment options for CRE infections.

## Review

### Introduction

The global spread of carbapenem-resistant *Enterobacteriaceae* (CRE) has become a major challenge in clinical and public health settings. Infections with CRE organisms that are multidrug-resistant (that is, non-susceptible to at least one antimicrobial in at least three antimicrobial classes), extensively drug-resistant (that is, non-susceptible to at least one antimicrobial from all but one or two antimicrobial classes), or pan-drug-resistant (that is, non-susceptible to all antimicrobial agents) are difficult to treat [1]. As a result, severe infections with CRE have significant morbidity, mortality, and health-care costs [2-4]. Most CRE have beta-lactamases belonging to the Ambler class A, B, and D [5]. Table 1 summarizes main examples from each class of carbapenemase-producing organisms.

**Table 1:** **Characteristics associated with carbapenemase**-**producing organisms commonly encountered in clinical practice**

**Class**	**Examples**	**Found commonly in**	**Epidemiology**	**Resistance phenotype**
A	KPC-2, KPC-3	*Klebsiella pneumoniae*, *Escherichia coli*	First isolated in US in 1996; now endemic in US, Colombia, Brazil, Argentina, Poland, Italy, Greece, Israel, and China [6]	All beta-lactams; often also fluoroquinolones and aminoglycosides [7]
B	NDM-1	*K. pneumoniae*, *E. coli*	First isolated in Sweden from a traveler previously hospitalized in New Delhi; large environmental reservoirs in India, Pakistan, Middle East, and the Balkans [8]; recent ERCP-related nosocomial outbreak associated with infected endoscopes reported in Chicago, IL [9]	Plasmids carry resistance genes to all beta-lactams, aminoglycosides, macrolides, rifampin, and trimethoprim- sulfamethoxazole [8]
D	OXA-48	*K. pneumoniae*	First identified in Turkey in 2003; multiple nosocomial outbreaks reported since then throughout the world [7]	All beta-lactams

ERCP, endoscopic retrograde cholangiopancreatography; KPC, *Klebsiella pneumoniae* carbapenemase; NDM, New Delhi metallo-beta-lactamase; OXA, oxacillin-hydrolyzing.

Carbapenems are no longer fully effective in the CRE epidemic. The paucity of novel antimicrobials in development escalates the antimicrobial resistance problem, severely reducing the available therapeutic choices. In this review, we will summarize the main treatment options used in clinical practice as well as the few antimicrobials currently in development. For issues related to the epidemiology, detection, and prevention of infections with CRE, the reader is referred to several excellent reviews published on this topic [6,10].

## Current treatment options for infections with carbapenem-resistant *Enterobacteriaceae*

The optimal treatment of infections with CRE is uncertain, as most data come from retrospective case series and anecdotal case reports; few prospective studies or randomized controlled trials are published on this topic. Since carbapenem-producing organisms are often resistant to other antimicrobial classes in addition to most beta-lactams, additional susceptibility testing to antimicrobials such as colistin, fosfomycin, tigecycline, aztreonam, and rifampin is needed [11,12]. Consultations from experts in the treatment of infections with multidrug-resistant organisms may also prove valuable in patient management. The following antimicrobial therapies have been used with various levels of success in the treatment of CRE infections.

## Colistin (polymyxin E)

Colistin (polymyxin E) is an old bactericidal antibiotic with cationic detergent properties. It disrupts the outer cell membrane of the Gram-negative bacilli by binding to the lipid A component of the lipopolysaccharide, causing leakage of cytoplasmic contents and bacterial cell death [13]. The antibacterial spectrum includes most of the *Enterobacteriaceae* (*Escherichia coli*, *Klebsiella*, *Salmonella*, *Shigella*, and *Enterobacter*), *Pseudomonas*, *Acinetobacter*, and *Stenotrophomonas* species. However, colistin is not active against *Burkholderia cepacia*, *Serratia marcescens*, *Moraxella catarrhalis*, pathogenic *Neisseria* spp, *Proteus* spp, *Providencia* spp, or *Morganella morganii*[14]. Colistin is also not active *in vitro* against anaerobes and aerobic Gram-positive cocci.

The ideal dose for colistin in the treatment of severe CRE infections is uncertain. In addition, significant confusion may arise because of differences in formulations between the intravenous (IV) product available in the US (colistin-based) and the one available in Europe and other regions (colistimethate sodium) (Table 2). Recent studies suggest that higher treatment doses [15] or an initial loading dose followed by higher maintenance dosing regimens may be needed for improved clinical outcomes, especially for infections with organisms with high minimum inhibitory concentrations (MICs) [16]. Specifically, for organisms with a colistin MIC of not more than 2 mg/L, some authors recommend a loading dose of 2.5 mg/kg given over a 2-hour infusion, followed by a maintenance dose of 3 mg/kg per day, based on population pharmacokinetic studies in critically ill patients [16]. Colistin monotherapy is not recommended for organisms with MICs to colistin of at least 4 mg/L [16]. Dalfino and colleagues [17], in their prospective cohort study of 25 critically ill patients with bacteremia or ventilator-associated pneumonia caused by CRE (*Klebsiella*) and other carbapenem-resistant bacteria (*Acinetobacter* and *Pseudomonas*), used a regimen of 9 million IU of colistimethate sodium loading dose (270 mg colistin base), followed by a maintenance dose of 4.5 million IU of colistimethate sodium (135 mg colistin base) every 12 hours in patients with normal renal function. For patients with underlying renal injury, the dosing interval was adjusted appropriately based on their renal clearance. The clinical cure achieved in this high-dose study was 82.1%, with a 17.8% rate of colistin-related acute kidney injury which was reversible within 10 days of discontinuing the drug. Of note, colistin monotherapy was administered to less than half of the patients in this study. Most patients received combination therapy with a carbapenem or aminoglycoside in addition to colistin, although only eight *Klebsiella pneumoniae* isolates were susceptible to gentamicin and none of the isolates was susceptible to carbapenems [17]. The colistin dosing strategy used by Dalfino and colleagues in this study of critically ill patients does seem to validate the recommendations from recent population pharmacokinetic analyses [18-20], suggesting that for severe infections in ICU patients the most effective bacterial killing is obtained with a loading dose, followed by higher overall maintenance doses given at extended intervals.

**Table 2:** **Dosing recommendations for colistin**-**based products available for parenteral use in critically ill patients**

**Region**	**Product type**^**a**^	**Dose recommended**
US	Colistin base activity	Loading dose: 270 mg colistin base
	(150 mg vials)	Maintenance dose: 135 mg colistin base every 12 hours
Other	Colistimethate sodium	Loading dose: 9 million IU
	(1 and 2 million IU vials)	Maintenance dose: 4.5 million IU every 12 hours

^a^30 mg colistin base activity = 1 million IU colistimethate sodium = approximately 80 mg colistimethate sodium [15,17]. These doses are based on normal renal function; renal adjustment is indicated for creatinine clearance of less than 50 mL/minute [17].

The importance of combination therapy is also suggested by several other retrospective studies. In a cohort of patients with bloodstream infections caused by *K. pneumoniae* carbapenemase (KPC)-producing organisms, none of the 14 patients treated with colistin in combination with one or more antimicrobials (tigecycline ± carbapenem ± gentamicin) died, whereas four of the seven patients treated with colistin alone died from their infection [21]. Similarly, colistin-polymyxin B combined with carbapenem had a mortality of 12.5% (1 out of 6) versus 66.7% (8 out of 12) in a study of patients with bacteremia caused by KPC-producing *K. pneumoniae* bacteremia [22]. Finally, in a large retrospective study of 125 patients with KPC-producing *K. pneumoniae* sepsis from three hospitals in Italy, the combination of colistin with tigecycline and extended-infusion meropenem (2 g IV infused over 3 hours every 8 hours) had the lowest mortality (13%) versus 50% mortality for those patients receiving colistin monotherapy [23].

The most common adverse event with colistin is nephrotoxicity, which can develop in up to half of the patients treated with high parenteral doses but which seems to be reversible in most cases [20,24]. Reports of resistance to colistin among KPC-producing *K. pneumoniae* strains [25,26], though rare, are concerning, especially for combination treatment regimens where colistin is intended as the major active component.

### Polymyxin B

Polymyxin B differs from colistin by one amino acid [27]. In contrast to colistin, however, it is administered as its active form and thus achieves higher plasma concentrations faster, making the need for a loading dose less stringent [16]. Polymyxin B is not cleared by the kidney and therefore does not require renal dose adjustment [28]. The clinical experience with polymyxin B in treatment of CRE infections is limited to small case series. Bergamasco and colleagues [29], in their description of a KPC-producing *K. pneumoniae* nosocomial outbreak among solid organ transplant patients, reported a survival rate of 67% (6 out of 9) for the patients treated with polymyxin B alone or in combination with tigecycline or carbapenem. These patients with pneumonia, bloodstream, urinary tract, or skin and soft tissue infections were given a polymyxin B dose of 25,000 or 15,000 IU/kg for a creatinine clearance of at least 50 mL/minute or less than 50 mL/minute, respectively. As is true for colistin, polymyxin B used in combination therapy for severe infections may be more effective, especially when one considers the possibility of resistance development during monotherapy. In this regard, Lee and colleagues [30] described the emergence of resistance to polymyxin B for three out of 12 patients treated with polymyxin B for their KPC-producing *K. pneumoniae* bloodstream infections; in contrast, none of the four patients treated with polymyxin B in combination with tigecycline developed resistance during therapy [30].

### Carbapenems

Carbapenems have been used, though counter-intuitively, in the treatment of infections with CRE, usually as the adjuvant component of a combination drug regimen. This strategy is potentially useful only when the MICs of the infecting carbapenem-resistant organisms are still relatively low (that is, not more than 4 to 8 mg/L) [31]. Thus, the MICs should always be determined and taken into account if carbapenems are contemplated as a potential treatment option. Bacterial killing for isolates with MICs of 4 mg/L is more likely with high-dose, prolonged-infusion regimens (that is, meropenem 2 g IV infused over 3 hours every 8 hours) [32]. The outcomes of carbapenem treatment in patients infected with multidrug-resistant Gram-negative organisms, including CRE, as reported anecdotally, in small case series or small retrospective clinical studies are summarized in the excellent review by Daikos and Markogiannakis [31]. A systematic review of 34 studies compiling a total of 298 patients treated for infections with KPC or metallo-beta-lactamase-producing *K. pneumoniae* found a combination regimen of at least two active drugs, one of which was a carbapenem, to be associated with the lowest failure rate (8%) compared with other regimens studied [10] (Table 3). As previously mentioned, Tumbarello and colleagues [23] found the triple-combination regimen of colistin, tigecycline, and meropenem to be associated with the highest odds of survival in their multicenter retrospective cohort study of 125 patients with KPC-producing *K. pneumoniae* bloodstream infections. Meropenem was administered as an extended infusion over at least 3 hours, at 2 g IV every 8 hours, with renal adjustment as needed. However, although more than 50% of the isolates in this study were fully resistant to meropenem (MIC of at least 16 mg/L), the vast majority of the isolates were susceptible to colistin (88% with MICs of not more than 2 mg/L) and tigecycline (91.2% with MICs of not more than 2 mg/L).

**Table 3:** **Treatment regimens and outcomes of various infections with carbapenemase**-**producing organisms reported in the literature**

**Infection**	**Pathogens**	**Number**	**Treatment**	**Mortality**	**Reference**
Bacteremia	KPC	125	Monotherapy	25/46 (54%)	[23]
	*K. pneumoniae*		Combination therapy	27/79 (34%)	
				7/23 (30%)	
			Colistin^a^ + tigecycline^b^	6/12 (50%)	
				2/16 (12%)	
			Tigecycline + gentamicin^c^		
			Colistin + tigecycline + meropenem^d^		
Bacteremia	KPC	41	Monotherapy	11/19 (58%)	[22]
	*K. pneumoniae*		Combination therapy^e^	2/15 (13%)	
				1/5 (20%)	
			Colistin + carbapenem	0/3 (0%)	
				0/2 (0%)	
			Tigecycline + carbapenem		
			Tigecycline + aminoglycoside		
Trauma ICU	KPC	26	Combination therapy	2/26 (8%)	[33]
VAP	*K. pneumoniae*				
Bacteremia			Tigecycline^f^ + gentamicin^g^ ± fosfomycin^h^		
			Tigecycline plus colistin^i^ ± fosfomycin		
Bacteremia	*Acinetobacter*	28	Monotherapy (colistin^j^)	2/5 (40%)	[17]
VAP	*Pseudomonas*		Combination therapy	3/14 (21 %)	
	KPC *K. pneumoniae*		Colistin + aminoglycoside		
			Colistin plus carbapenem		
ICU	KPC *K. pneumoniae*	11	Combination therapy	2/11 (18%)	[34]
VAP ± bacteremia					
UTI, wound			Fosfomycin^k^ + colistin		
			Fosfomycin + gentamicin		
			Fosfomycin + piperacillin/tazobactam		
ICU bacteremia	KPC *K. pneumoniae*	48	Combination therapy	18/48 (37.5%)	[35]
VAP	*Pseudomonas*		Fosfomycin^l^ + colistin		
			Fosfomycin + tigecycline		
UTI	KPC *K. pneumoniae*	21	Gentamicin	0/7 (0%)	[36]
			Other	0/7 (0%)	
			Doxycycline		
			Ciprofloxacin		
			Nitrofurantoin		
			Colistin		
UTI (including colonization)	CRE	136	Treatment received^m^	9/136 (7%)	[37]
			Polymyxin B		
			Tigecycline		
			Aminoglycoside		
			No treatment		

^a^Initial loading dose, followed by 6 to 9 million IU/day; ^b^initial loading dose followed by 100 to 200 mg/day; ^c^4 to 5 mg/kg per day; ^d^2 g every 8 hours infused over at least 3 hours; ^e^various other combinations used infrequently; the other patient who died on combination therapy received carbapenem-fluoroquinolone; ^f^100 mg intravenous (IV) every 12 hours; ^g^5 to 7 mg/kg per day; ^h^4.5 million IU every 12 hours; ^i^3 g IV every 8 hours; ^j^9 million IU loading dose, followed by 4.5 million IU every 12 hours if normal renal function; ^k^4 g IV every 6 hours; ^l^IV dose up to 24 g/day; ^m^rates of microbiological cure were 88% in the aminoglycoside group (n = 41), 64% in the polymyxin B group (n = 25), 43% in the tigecycline group (n = 21), and 36% in the no treatment group (n = 69). CRE, carbapenem-resistant *Enterobacteriaceae*; KPC, *Klebsiella pneumoniae* carbapenemase; UTI, urinary tract infection; VAP, ventilator-associated pneumonia.

Recently, a double-carbapenem combination (ertapenem-doripenem) has been proposed as a potential treatment strategy for KPC-producing bacteria [38,39]. Data come from *in vitro* experiments on a murine animal model [38] as well as *in vivo*. Regarding the latter, three patients with bacteremia or urinary tract infection (UTI) caused by pan-resistant KPC-producing *K. pneumoniae*[39] and one ICU patient with bacteremia and sepsis caused by colistin-resistant KPC-producing *K. pneumoniae* were reported to have been successfully treated with a double-carbapenem combination [40]. Most recently, Karaiskos and colleagues [41] reported treating 14 patients with bacteremias and UTIs, including two patients with septic shock caused by KPC-producing *K. pneumoniae* with double-carbapenems therapy, as follows: 1 g ertapenem IV daily, followed 1 hour later by meropenem at 2 g every 8 hours infused over 3 hours. All treated patients experienced clinical and microbiological cure at 1-month follow-up, although four patients experienced a recurrence of their UTI [41]. Nevertheless, since the clinical experience with this salvage therapy is still limited, concerns for promoting further carbapenem resistance remain [33], and the MICs of many carbapenem-producing organisms are sufficiently high to render carbapenems ineffective, this treatment strategy is not routinely recommended for clinical practice at the present time.

### Tigecycline

Tigecycline has been shown to have *in vitro* activity against multidrug-resistant *Enterobacteriaceae* isolates [42]. Tigecycline has been used in the treatment of infections with CRE primarily as an adjuvant drug in combination therapy (Table 3) [43-45]. However, the clinical experience with tigecycline has been somewhat disappointing, especially for severe infections such as bloodstream infections or nosocomial pneumonias, for which the drug does not have US Food and Drug Administration (FDA) approval. For example, Kontopidou and colleagues [46], in their study of 127 ICU patients with bacteremias or ventilator-associated pneumonias caused by carbapenem-resistant *K. pneumoniae*, found that patients treated with tigecycline, especially as monotherapy (at doses of 100 to 200 mg/day), had the highest failure rates compared with other drug combinations. Most patients treated with tigecycline in this cohort had an MIC of 2 μg/mL (which is the cutoff for susceptibility) and severe infections with high Acute Physiology and Chronic Health Evaluation II scores, which may explain why tigecycline was ineffective [46]. Post-approval meta-analyses have shown that tigecycline had lower cure rates and higher mortality compared with other treatment regimens in pooled randomized controlled trials of various infectious syndromes [47-49]. When evaluated in a randomized controlled trial of hospital-acquired pneumonia, tigecycline plus ceftazidime was inferior to vancomycin and imipenem-cilastatin for the treatment of ventilator-associated pneumonia [50]. The problem may be related to the low plasma serum concentrations achieved by the dose recommended by the manufacturer (100 mg loading dose followed by a maintenance dose of 50 mg every 12 hours), which is likely ineffective against pathogens with an MIC of between 0.4 and 1 mg/L. Higher doses have been used in clinical practice [51]. In fact, a recent phase 2 randomized controlled trial of patients with hospital-acquired pneumonia studied higher doses of tigecycline (150 mg loading followed by 75 mg every 12 hours, and 200 mg loading followed by 100 mg maintenance dose every 12 hours) versus imipenem/cilastatin. Clinical cure rates were the highest in the arm with the highest dose regimen of tigecycline, whereas the safety profile was similar to that of the lower dose regimens [52]. Nevertheless, in 2010, the FDA added a warning regarding the risk of increased mortality with tigecycline treatment, especially for non-approved indications such as hospital- or ventilator-associated pneumonias (found at [53]). This safety concern was upgraded to a stronger Boxed Warning in 2013, after analysis of 10 clinical trials of tigecycline use for FDA-approved indications, including trials conducted after drug approval, still showed a higher (0.6%) risk of death for patients treated with tigecycline versus other antimicrobials (found at [54]). As a result, many clinicians have chosen tigecycline-based regimens only when other therapies were not available. The low concentration of tigecycline in the urine further limits the use of this antimicrobial for the treatment of UTIs. Unless more compelling evidence of improved clinical outcomes in well-designed studies of high-dose tigecycline becomes available, tigecycline monotherapy is not routinely recommended for severe infections such as bacteremia or hospital-acquired pneumonia.

### Fosfomycin

Fosfomycin is another old broad-spectrum antibiotic that inhibits bacterial cell wall synthesis and has *in vitro* activity against CRE [12,55]. The oral formulation achieves high concentrations in the urine and is usually effective in the treatment of non-complicated UTIs [56]. The IV formulation (fosfomycin disodium) is not available in the US and other countries, although it has been used successfully in Greece, mostly as an adjuvant drug in combination therapies [57]. For example, a study of 11 critically ill patients with nosocomial infections caused by KPC *K. pneumoniae* were treated with IV fosfomycin (2 to 4 g every 6 hours) in combination with colistin (n = 6), gentamicin (n = 3), and piperacillin/tazobactam (n = 1). All patients were reported to have good treatment-related microbiological and clinical outcomes, while the all-cause hospital mortality was 18.2% (two patients) [34]. The emergence of resistance to fosfomycin during therapy for bacteremia with KPC *K. pneumoniae* has been reported and is especially concerning since fosfomycin was used as an adjunct in combination therapy in these cases [58]. Recently, parenteral fosfomycin administered in combination with colistin or tigecycline was studied in a prospective observational multicenter trial in 11 ICUs in Europe. In total, 41 patients with bacteremia or ventilator-associated pneumonia caused by carbapenemase-producing *K. pneumoniae* were treated with a median dose of 24 g of fosfomycin per day for a total of 14 days. Microbiological cure was reported in 56.5% of cases, with an all-cause 28-day mortality of 43.5% and emergence of resistance in three patients [35].

## Antimicrobials in development

Several parenteral antimicrobial therapies are currently under investigation for the treatment of multidrug-resistant Gram-negative infections, including CRE. Ceftazidime-avibactam (a new beta-lactamase inhibitor) is active against extended-spectrum beta-lactamase-producing organisms, some resistant *Pseudomonas aeruginosa* strains, and CRE of the KPC type, but not against metallo-beta-lactamases such as New Delhi metallo-beta-lactamase and verona integron-encoded metallo-beta-lactamase. It is currently undergoing phase 3 studies for complicated UTI and intra-abdominal infections [59]. Ceftaroline-avibactam, entering phase 3 trials, is similarly active against KPC-producing strains, but not against *P. aeruginosa* or other metallo-beta-lactamase-producing organisms. Neither one of these drugs in development has activity against *Acinetobacter* species [6,59]. Imipenem in combination with another novel beta-lactamase inhibitor, MK-7655, appears active *in vitro* against serine carbapenemase-producing organisms and against *P. aeruginosa*, but not against metallo-carbapenemase-producing organisms or *Acinetobacter baumannii*[59]. Plazomycin (ACHN-490), a new aminoglycoside currently in development, has activity against isolate-producing KPC enzymes and does not seem susceptible to the same resistance mechanisms present in older aminoglycosides, although it does not have activity against strains that harbor 16S ribosomal methylases. It has completed phase 2 trials [60]. Biapenem/RPX7009 (Carbavance; Rempex Pharmaceuticals, Inc., San Diego, CA, USA), a carbapenem combined with a novel boronate inhibitor, currently in phase 1 trials, appears active *in vitro* against KPC-producing organisms and other class A carbapenemases, including resistant *Pseudomonas* and *Acinetobacter* strains, although it is not active against class B and D carbapenemases [6,61]. Eravacycline is a novel tetracycline that is not susceptible to efflux resistance mechanisms or to the protection of the ribosomal target that renders older tetracyclines ineffective. It has *in vitro* activity against KPC-producing bacteria but not against non-fermenters [62].

## Conclusions

None of the antimicrobials currently in development has activity against the entire spectrum of carbapenemase-producing Gram-negative bacteria. The mortality associated with the failure rates from the current salvage therapies highlighted above is disconcerting. The treatment of serious infections with CRE remains an enormous challenge. A concerted global commitment to the intelligent use of antimicrobials, better antibiotic stewardship, the implementation of effective infection control strategies, and the development of more effective therapies are desperately needed.

## Note

This article is part of a series on *Antibiotic resistance in the ICU*, edited by Steven Opal. Other articles in this series can be found at http://ccforum.com/series/antibioticresistance.

## Abbreviations

CRE: Carbapenem-resistant *Enterobacteriaceae*; FDA: US Food and Drug Administration; IV: Intravenous; KPC: *Klebsiella pneumoniae* carbapenemase; MIC: Minimum inhibitory concentration; UTI: Urinary tract infection.

## Competing interests

The authors declare that they have no competing interests.
